# Blonanserin vs risperidone in Japanese patients with schizophrenia: A post hoc analysis of a phase 3, 8‐week, multicenter, double‐blind, randomized controlled study

**DOI:** 10.1002/npr2.12089

**Published:** 2019-12-01

**Authors:** Philip D. Harvey, Hiroshi Nakamura, Sadanori Miura

**Affiliations:** ^1^ Leonard M. Miller Professor of Psychiatry and Behavioral Sciences University of Miami, Miller School of Medicine Miami FL USA; ^2^ Medical Affairs Sumitomo Dainippon Pharma Co., Ltd Tokyo Japan; ^3^ Kitasato University School of Medicine Kanagawa Japan

**Keywords:** antipsychotic agents, blonanserin, randomized controlled trial, risperidone, schizophrenia

## Abstract

**Objective:**

To report the efficacy and safety of blonanserin in patients with schizophrenia compared with risperidone in a Japanese multicenter, randomized, double‐blind study based on post hoc sensitivity analysis in addition to the previous results reported by Miura and discuss the current approaches for schizophrenia treatment.

**Methods:**

Of 302 patients randomized, 156 received blonanserin (8‐24 mg/d) and 145 received risperidone (2‐6 mg/d) for 8 weeks. Efficacy variables included the Positive and Negative Syndrome Scale (PANSS) total score for the primary outcome, PANSS subscale, Brief Psychiatric Rating Scale (BPRS), and Clinical Global Impression‐Improvement (CGI‐I) for secondary outcomes. Safety variables included treatment‐emergent adverse events, Drug Induced Extrapyramidal Symptoms Scale scores, laboratory data, vital signs, electrocardiogram, etc

**Results:**

Blonanserin was not inferior to risperidone in the change in PANSS total score at a non‐inferior margin of −7 (intergroup difference, −0.46; 95% CI, −4.40 to 3.48). Post hoc analyses wholly supported the primary result. No major difference was found in the changes in BPRS scores and the improvement rate on CGI‐I between the drugs. The incidence of adverse events was similar in the two drugs. Blonanserin was associated with a lower risk of prolactin increase, weight gain, and orthostatic hypotension compared with risperidone. However, blonanserin was associated with a higher incidence of akathisia and excitability compared with risperidone. Most of the adverse events were mild to moderate in severity with no specific events of predominant high severity in the both drugs.

**Conclusions:**

Blonanserin exerted the similar efficacy to risperidone in both positive and negative symptoms in schizophrenia with a lower risk of prolactin increase, weight gain, and orthostatic hypotension compared with risperidone. Blonanserin will serve as a favorable treatment option for schizophrenia in daily clinical practice.

## Abstract 2

Blonanserin exerted the similar efficacy in both positive and negative symptoms in schizophrenia and similar well‐tolerable safety profiles with minor variation compared with risperidone. Further investigation is needed to clarify the potential benefit of blonanserin in the context of the latest schizophrenia treatment.

## Graphical abstract

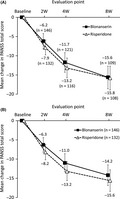

## 1 INTRODUCTION

Schizophrenia is a complex disorder with three sets symptoms: positive symptoms such as hallucination or delusion, negative symptoms including low motivation, and cognitive impairment characterized by difficulties in executive functions or information processing speed. Conventional treatment of schizophrenia has targeted the psychiatric symptoms in the acute phase and successfully treated the positive symptoms with first‐ and second‐generation antipsychotics (FGAs and SGAs). Second‐generation antipsychotics not only improve positive symptoms similar to FGAs but also relieve negative symptoms better than FGAs^1^, ^2^ with a small effect size and with a low incidence of extrapyramidal symptoms and hyperprolactinemia. And thus, SGAs are recommended as first‐line therapy in current treatment guidelines for schizophrenia.^3^ As noted above, the overall therapeutic effect of SGAs against negative symptoms is considered limited.^4^

Blonanserin is a relatively new SGA that has been approved in Japan (2008), Korea (2009), and China (2017) with an indication for schizophrenia, which has high receptor selectivity as a potent antagonist of the dopamine D_2_, D_3_, and serotonin 5‐HT_2A_ receptors with low affinity for the dopamine D_1_, serotonin 5‐HT_2C_, adrenaline α1, histamine H_1_, and muscarinic M_1_ receptors. Due to its receptor‐binding profiles, blonanserin is expected to improve positive and negative symptoms, while suppressing extrapyramidal side effects that were a serious problem in FGAs. Previous reports on randomized controlled trials showed similar efficacy of blonanserin in various psychiatric symptoms in schizophrenia compared with haloperidol or risperidone.^5^, ^6^

The comparator, risperidone, is one of the most widely used SGAs. It has high affinity for serotonin 5‐HT_2A_ and 5‐HT_7_, dopamine D_2_, adrenergic α_1_ and α_2_ receptors and a moderate affinity for histamine H_1_ receptors.

Miura had already reported the results of the phase 3 study comparing blonanserin and risperidone in Japanese patients with schizophrenia (Published in Japanese only).^6^ This study was conducted in response to Japanese regulatory requirements for approval of blonanserin; however, recent guidelines^7^ recommend sensitivity analyses to confirm primary evidence of the studies with non‐negligible missing data. Therefore, we added mixed model for repeated measures (MMRM) analysis, completer analysis, and responder rate analysis to the previous last observed carried forward (LOCF) approach for the primary endpoint of the study and assessed the robustness of the result. Here, we report the efficacy and safety of blonanserin in patients with schizophrenia vs risperidone based on the previous and new results, and discuss the current approaches for treatment of schizophrenia.

## 2 METHODS

Most of the methods used in the study were previously described.^6^ However, since the previous study was published in the Japanese language, the details of the methods for the entire study are shown below.

### 2.1 Design

This randomized, double‐blind, multicenter study was conducted from August 2003 through November 2004 at 59 medical institutions in Japan. The institutional review board at each study site approved the study protocol. The study was conducted in compliance with the Good Clinical Practice (GCP) and the applicable regulatory requirements. Written informed consent was obtained from all patients, or their legal representatives if patients were unable to give consent or were younger than 20 years old before any study procedures were performed.

### 2.2 Participants

The inclusion criteria were as follows: at least 15 years of age, met the F20 schizophrenia criteria of the International Classification of Diseases (ICD) 10 (Diagnostic Criteria for Research) and had a Positive and Negative Syndrome Scale (PANSS) total score between 60 and 120. The exclusion criteria included the prominent state of excitement or stupor, personality disintegration, being refractory to treatment, previous nonresponse to risperidone at doses up to 6 mg/d, use of any long‐acting antipsychotic drug within 28 days before the initial study treatment, a history of neuroleptic malignant syndrome or water intoxication, debilitation by dehydration or malnutrition, high risk of self‐harm or suicide attempt, diagnosed or suspected diabetes mellitus, and meeting the guidance of contraindication for careful administration of risperidone.

### 2.3 Study procedure

Patients were randomized into blonanserin or risperidone at a ratio of 1:1 using computer‐generated random numbers with block randomization (six patients per block). They were switched from previous treatment without a taper of the prior medication. They received study drugs orally twice daily, after breakfast and evening meal, for 8 weeks. Blinding was maintained with a double‐dummy design. Blonanserin was administered as 2‐ or 4‐mg tablet, started from 8 mg/d, and could be adjusted thereafter between 8 and 24 mg/d, which was the same manner as that in the preceding haloperidol‐controlled study.^5^ Risperidone was given as 1‐ or 2‐mg tablet, started from 2 mg/d and could be adjusted thereafter between 2 and 6 mg/d according to the agreement made with Japanese authority.^6^ The range is in line with the current Japanese label of risperidone recommending 2‐6 mg/d and the US FDA current label not recommending doses above 6 mg/d, although both labels also allow a higher dose of risperidone. Several clinical studies indicated that risperidone at a dose higher than 6 mg/d might induce side effects such as extrapyramidal symptoms.^8^, ^9^ The dose was increased by 4 mg/d for blonanserin and 1 mg/d for risperidone if the change from baseline in the Brief Psychiatric Rating Scale (BPRS, see Section 2.4) total score at the study visit was −5 or higher and no major safety concern was observed. If a major safety concern was found, a dose reduction was allowed. Prohibited concomitant therapy were antipsychotics including Vegetamin^®^ and levomepromazine, carbamazepine, methamphetamine hydrochloride, epinephrine, CYP3A4 inhibitors, and electroconvulsive therapy. Prophylactic antiparkinsonian medications were prohibited. Prior antiparkinsonian drugs were discontinued by 2 weeks after the initiation of the study treatment, but its concomitant use was allowed if extrapyramidal symptoms worsened or newly occurred.

### 2.4 Variables

The primary variable was the change in PANSS total score from baseline at the end of study. The efficacy variables were assessed with the PANSS Japanese edition,^10^ the BPRS Japanese edition,^11^ and the Clinical Global Impressions‐Improvement (CGI‐I). The CGI‐I is a physician‐rated scale to assess the general change from baseline in the patient's condition. The change is rated on a seven‐rank scale of very much improved, much improved, minimally improved, no change, minimally worse, much worse, and very much worse compared with baseline, or otherwise reported as not evaluable. The outcome was adjusted for prior antipsychotics to exclude their effect on the efficacy evaluation for this study. The PANSS was assessed at baseline and after 2, 4, and 8 weeks of study treatment (ie, Weeks 2, 4, and 8) or at study discontinuation, and the CGI‐I and BPRS were assessed at baseline (CGI‐I was assessed for on‐treatment patients only), Weeks 1, 2, 3, 4, 6, and 8 or at study discontinuation.

Safety variables were treatment‐emergent adverse events, the Drug Induced Extra Pyramidal Symptoms Scale (DIEPSS) scores,^12^ laboratory data (hematology, blood chemistry, and urinalysis), vital signs (blood pressure, pulse rate, and body temperature), weight, 12‐lead electrocardiography (ECG) findings at rest, and electroencephalography (EEG) findings. A central reading was performed for ECG findings. Adverse events were coded and classified into the system organ classes and preferred terms of the Medical Dictionary for Regulatory Activities Japanese version (MedDRA/J) 7.0 and translated into English. A relationship with the study drug was classified as definitely related, probably related, possibly related, not related, and unknown.

### 2.5 Statistical analysis

Statistical analyses were performed using SAS version 8.2 (SAS Institute Japan Ltd.). The statistical population consisted of all enrolled patients without GCP violation who were randomized and treated with at least one dose of the study drug (safety analysis population) and had at least one post‐baseline efficacy assessment (efficacy analysis population). The two‐sided 95% confidence interval (CI) of the intergroup difference was calculated for the PANSS scores, the BPRS scores, and the percentage of patients rated as very much improved or much improved on the CGI‐I. Noninferiority of blonanserin to risperidone was to be confirmed if the lower bound of the 95% CI of the intergroup difference in the primary efficacy variable, the change in the PANSS total score from baseline at the end of study, exceeded a predefined noninferiority threshold of −7. The threshold was based on the results from Japanese and non‐Japanese clinical studies of risperidone.^13^, ^14^ The tests were not adjusted for multiplicity. Missing data at Week 8 were imputed with LOCF strategies in the previous report by Miura^6^. In the present report, we newly conducted the following post hoc sensitivity analyses for PANSS total scores; MMRM analysis, responder (ie, patients with greater than or equal to 20% or 30% improvement from baseline) rate analysis, and completer (ie, patients completed the protocol treatment) analysis. The incidence of adverse events was calculated for each group. Laboratory data, vital signs, weight, ECG parameters, and EEG parameters were analyzed for change from baseline and the incidence of abnormal change by treatment group and evaluation point. The intergroup difference in the change in PANSS total score of 0 and a standard deviation of 20 was assumed.^5^, ^13^ A total of 260 patients (130 per group) were required to provide a power of 80% to establish noninferiority. Assuming that some patients would be excluded from the analysis, a sample size of 300 patients (150 per group) was planned.

## 3 RESULTS

### 3.1 Patient characteristics

**Figure 1:**
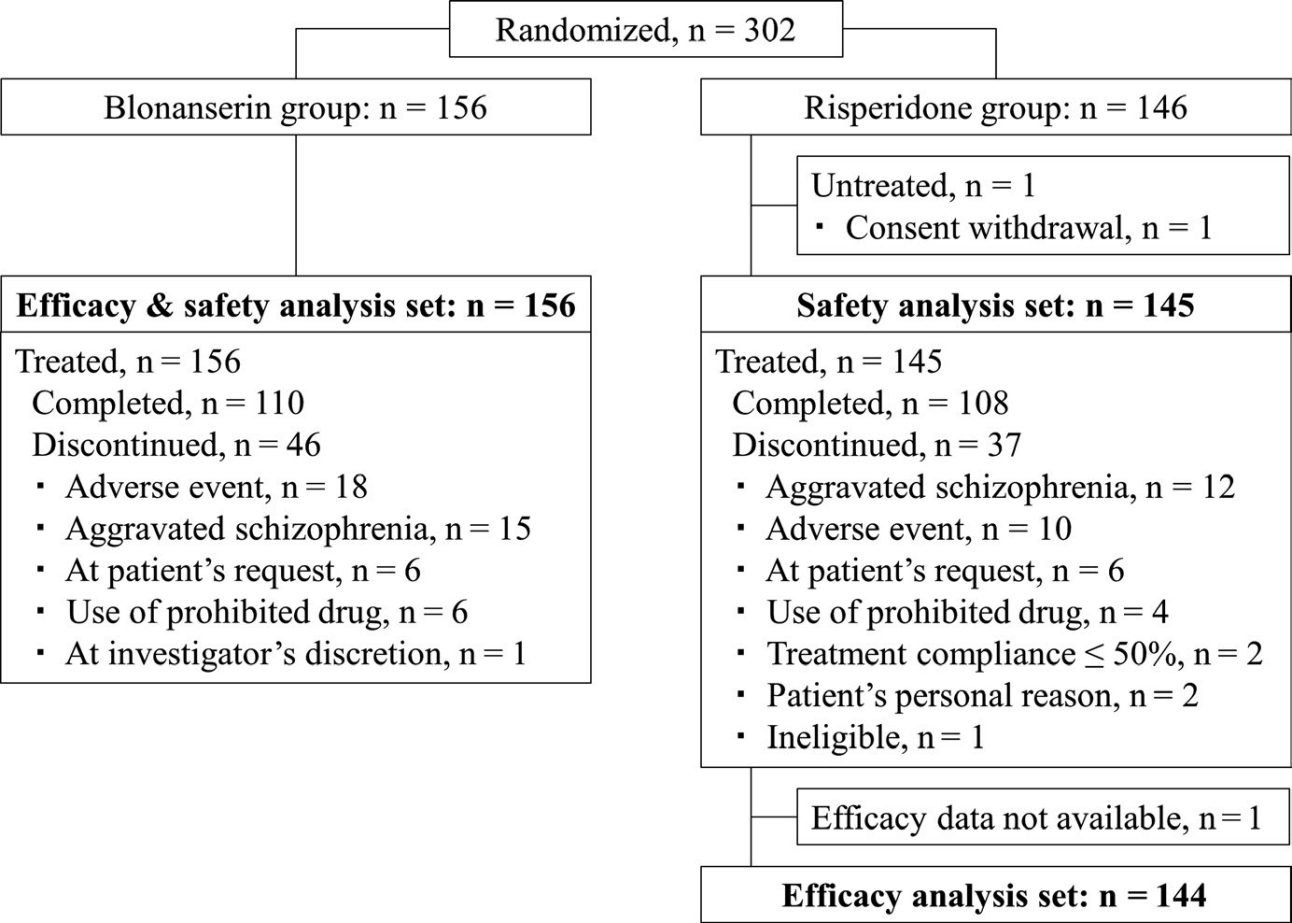
CONSORT diagram for study flow. Patient disposition and analysis populations. Patients who discontinued the study for more than 1 reasons were counted for each category

Of 302 patients randomized, 301 received study treatment (Figure 1). The withdrawal rate was similar in the two groups: 29.5% in the blonanserin group and 25.5% in the risperidone group. All the 301 treated patients were included in the safety analysis (156 in the blonanserin group and 145 in the risperidone group). Since one patient in the risperidone group died during the study and thus had no post‐baseline efficacy data, the remaining 300 patients (156 in the blonanserin group and 144 in the risperidone group) were included in the efficacy analysis.

In each group, the number of men was slightly higher than that of women, and the mean age was about 45 years (Table 1). Duration of schizophrenia was 5 years or longer in about 80% of the patients. The most prominent schizophrenia subtypes were paranoid, hebephrenic, and residual schizophrenia according to the ICD‐10. The mean baseline PANSS total score was close to 90, and the negative symptoms were prominent in more than 75% of the patients. Most patients were receiving antipsychotics, and almost half of the patients were receiving antiparkinsonian drugs. In patients with prior antipsychotics, no notable intergroup difference was found in the baseline improvement rate on the CGI‐I (Table S1).

**Table 1:** Baseline patient characteristics

Category	Blonanserin N = 156	Risperidone N = 144
Sex, n (%)		
Male	88 (56.4)	75 (52.1)
Age (y), mean ± SD	45.0 ± 14.8	46.0 ± 14.5
Duration of disease (y), n (%)		
<1	9 (5.9)	9 (6.6)
≥1, <2	4 (2.6)	4 (2.9)
≥2, <3	6 (3.9)	3 (2.2)
≥3, <5	13 (8.5)	12 (8.8)
≥5, <10	24 (15.7)	28 (20.6)
≥10	97 (63.4)	80 (58.8)
Unknown^a^	3 (—)	8 (—)
Disease type by ICD‐10, n (%)		
Paranoid	65 (41.7)	65 (45.1)
Residual	36 (23.1)	27 (18.8)
Hebephrenic	32 (20.5)	34 (23.6)
Undifferentiated	11 (7.1)	13 (9.0)
Catatonic	9 (5.8)	5 (3.5)
Simple	2 (1.3)	0 (0.0)
Unspecified	1 (0.6)	0 (0.0)
Post‐schizophrenic depression	0 (0.0)	0 (0.0)
Other	0 (0.0)	0 (0.0)
Disease type by DMS‐IV, n (%)		
Paranoid	65 (41.7)	65 (45.1)
Residual	37 (23.7)	30 (20.8)
Disorganized	33 (21.2)	29 (20.1)
Undifferentiated	12 (7.7)	15 (10.4)
Catatonic	9 (5.8)	5 (3.5)
Others	0 (0.0)	0 (0.0)
Use of prior antipsychotics, n (%)		
Yes	138 (88.5)	128 (88.9)
Use of prior antiparkinsonian drugs, n (%)		
Yes	86 (55.1)	77 (53.5)
PANSS total score, mean ± SD	87.1 ± 14.7	86.7 ± 15.3
Dominance in PANSS, n (%)		
Negative symptoms	120 (76.9)	109 (75.7)
Positive symptoms	30 (19.2)	31 (21.5)
No prominence	6 (3.8)	4 (2.8)

Note

Adapted from Miura S. 2008, Table 3.

Abbreviations: —, not tested or calculated; DSM‐IV, Diagnostic and Statistical Manual of Mental Disorders, 4th edition; ICD‐10, International Classification of Diseases 10; PANSS, Positive and Negative Syndrome Scale; SD, standard deviation.

a The percentage was not calculated for "unknown."

### 3.2 Efficacy

#### 3.2.1 Primary variable

The PANSS total score was reduced from baseline to the end of the study in each group, and the change from baseline was similar in the 2 groups: −11.05 (standard deviation [SD], 17.27) in the blonanserin group and −11.51 (SD, 17.38) in the risperidone group (Table 2). The MMRM model showed the comparable results: the estimated changes at the end of study from baseline were −14.2 (standard error [SE], 1.38) in the blonanserin group and −15.6 (SE, 1.43) in the risperidone group (Table 2). Since the lower 95% CI bound of the intergroup difference in the change exceeded a predefined noninferiority threshold of −7, blonanserin was not inferior to risperidone.

**Table 2:** Change from baseline in PANSS total score at the end of study

population method	Group	N	Baseline, mean ± SD	Change from baseline		Treatment difference	
				Estimate (SE)	95% CI	Estimate (SE)	95% CI
FAS	Blonanserin	156	87.1 ± 14.91	−11.0 (1.38)	−13.8, −8.4	−0.5 (2.00)	−4.4, 3.5
LOCF	Risperidone	144	86.0 ± 15.03	−11.5 (1.45)	−14.3, −8.7		
FAS	Blonanserin	146		−14.2 (1.38)	−16.9, −11.5	−1.4 (1.97)	−5.3, 2.5
MMRM	Risperidone	132		−15.6 (1.43)	−18.4, −12.8		
Completer	Blonanserin	110	86.6 ± 14.97	−15.5 (1.49)	−18.5, −12.6	−0.3 (2.15)	−4.5, 4.0
LOCF	Risperidone	108	86.1 ± 14.44	−15.8 (1.55)	−18.8, −12.7		
Completer	Blonanserin	110		−15.6 (1.49)	−18.5, −12.6	−0.5 (2.10)	−4.6, 3.6
MMRM	Risperidone	108		−16.1 (1.51)	−19.1, −13.1		

Note

Adapted from Miura S. 2008, Table 4.

Abbreviations: CI, confidence intervals; FAS, full analysis set; LOCF, last observation carried forward; MMRM, mixed model for repeated measures; PANSS, Positive and Negative Syndrome Scale; SD, standard deviation; SE, standard errors.

Completer analysis confirmed the primary result: lower limit of 95% CI of intergroup difference was −4.5 and −4.6 for LOCF and MMRM analysis, respectively (Table 2).

#### 3.2.2 Secondary variables

##### PANSS

**Figure 2:**
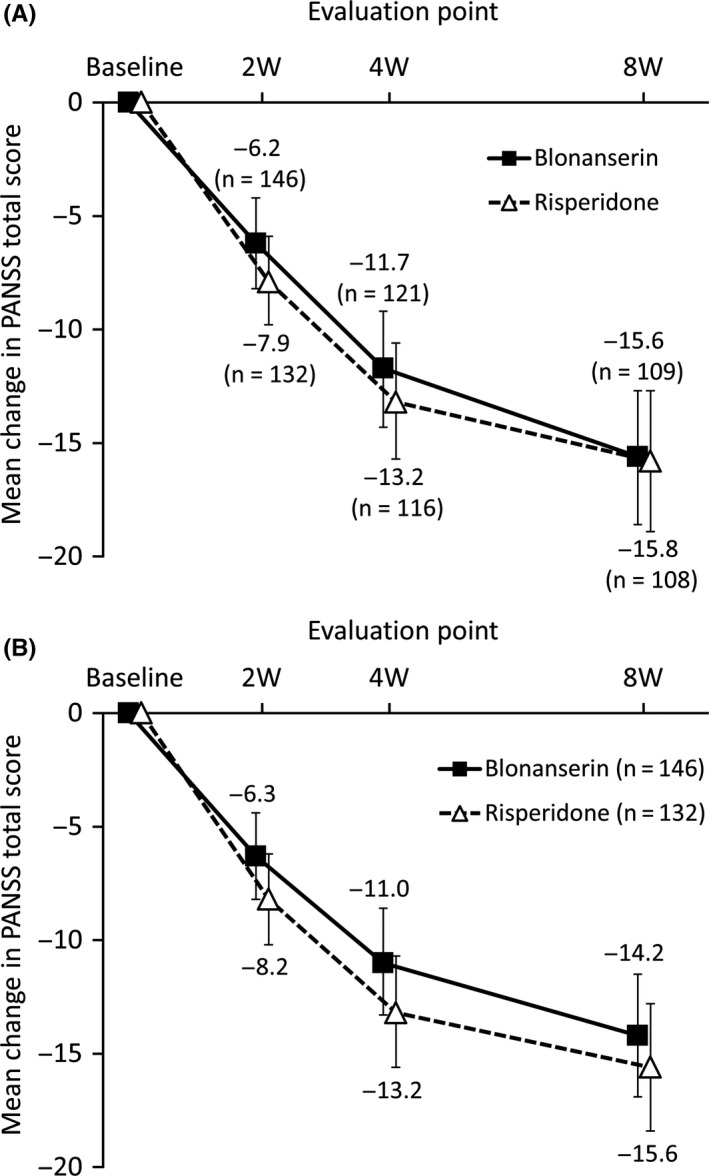
Time courses of change in Positive and Negative Syndrome Scale total score. Based on: A, Observed case; B, mixed model for repeated measures

The 2 groups showed the similar time course of PANSS total scores, which was lower than baseline at and after Week 2 (Figure 2A). Similarly, the total scores declined during the time course in MMRM analysis for both groups (Figure 2B). Responder rate of PANSS total score was similar in both groups as well (Table 3). Completer analysis also showed similar results in PANSS responder rate (Table 3). Besides, each PANSS subscale score (positive, negative, and general psychopathology subscales) decreased in both groups, and the change from baseline at the end of study was similar in the two groups for each subscale (Table 4).

**Table 3:** Positive and Negative Syndrome Scale Total score responders

Population	Improvement from baseline	Blonanserin	Risperidone
FAS		N = 156	N = 144
	≥20%, n (%)	72 (46.2)	71 (49.3)
	≥30%, n (%)	52 (33.3)	46 (31.9)
Completers		N = 110	N = 108
	≥20%, n (%)	64 (58.2)	65 (60.2)
	≥30%, n (%)	46 (41.8)	45 (41.7)

Note

Responders were defined as patients having a greater than or equal to 20% and 30% improvement from baseline.

Improvements are calculated as ([post‐baseline value − 30] − [baseline value − 30]) × 100/ (baseline value − 30).

Abbreviation: FAS, full analysis set.

**Table 4:** Comparisons of change in Positive and Negative Syndrome Scale subscale score at the end of study

Subscale	Group	N	Baseline	Change from baseline	
			Mean ± SD	Mean ± SD	95% CI
Positive subscale	Blonanserin	156	18.8 ± 5.2	−2.5 ± 5.5	−3.4, −1.6
	Risperidone	144	19.0 ± 6.2	−3.1 ± 5.9	−4.0, −2.1
Negative subscale	Blonanserin	156	24.3 ± 5.7	−3.4 ± 4.6	−4.2, −2.7
	Risperidone	144	24.6 ± 5.8	−3.0 ± 4.3	−3.7, −2.3
General psychopathology subscale	Blonanserin	156	44.1 ± 8.1	−5.1 ± 9.1	−6.6, −3.7
	Risperidone	144	43.1 ± 7.9	−5.5 ± 9.1	−7.0, −3.9

Note

Adapted from Miura S. 2008, Table 5.

Abbreviations: CI, confidence interval; PANSS, Positive and Negative Syndrome Scale; SD, standard deviation.

##### BPRS

Similarly, the two groups showed a similar time course of BPRS total score, which was lower than baseline at and after Week 1. No major difference was found in the change in BPRS total score or cluster scores at the end of the study between groups: −7.2 in the blonanserin group and −7.4 in the risperidone group (Table S2).

##### Clinical Global Impression

The improvement rate increased with time in each group and was similar at Week 8 in the two groups (Table S3). The improvement rate on the CGI‐I at the end of study was similar in the groups: 51.0% (79/155 patients) in the blonanserin group and 56.6% (81/143 patients) in the risperidone group (Table S4).

### 3.3 Safety

The mean average dose was 16.3 mg/d (SD, 6.2) for blonanserin and 4.0 mg/d (SD, 1.5) for risperidone at the end of study.

#### 3.3.1 Adverse events

Almost all patients experienced adverse events (Table 5). No noticeable intergroup difference was found in the incidence of adverse events. One patient in the risperidone group died (completed suicide). Other serious adverse events occurred in 3 patients of the blonanserin group (persecutory delusion/insomnia, suicide attempt, intentional overdose in one patient each) and one patient of the risperidone group (auditory hallucinations). All serious adverse events except the completed suicide in the risperidone group were considered by the local site investigators to be unrelated to the study drug.

**Table 5:** Summary of adverse events

	Blonanserin (N = 156)	Risperidone (N = 145)
	n (%)	n (%)
Adverse events	153 (98.1)	143 (98.6)
Adverse events leading to treatment discontinuation	33 (21.2)	22 (15.2)
Death	0	1 (0.7)
Serious adverse events	3 (1.9)	2 (1.4)
Adverse drug reactions	148 (94.9)	142 (97.9)
Patients with extrapyramidal adverse events^a^	104 (66.7)	89 (61.4)

Note

Adapted from Miura S. 2008, Table 10.

a Akathisia was included in the extrapyramidal adverse events. The extrapyramidal adverse events were oculogyration, dysphagia, salivary hypersecretion, asthenia, difficulty in walking, gait abnormal, corneal reflex decreased, posture abnormal, musculoskeletal stiffness, akathisia, bradykinesia, dysarthria, dyskinesia, dyslalia, dystonia, hypokinesia, speech disorder, tremor, and parkinsonian gait.

The most common adverse event in the blonanserin group was increase in blood prolactin, followed by insomnia, bradykinesia, tremor, akathisia, and somnolence (Table 6). The most common adverse event in the risperidone group was increase in blood prolactin, followed by bradykinesia, insomnia, tremor, malaise, and somnolence. Blonanserin was associated with a lower incidence of hyperprolactinemia, increase in blood prolactin, gamma glutamyl transpeptidase increase, weight increase, increased appetite, and orthostatic hypotension. Risperidone was associated with a lower incidence of akathisia, excitability, and pruritus.

**Table 6:** Incidence of adverse events (≥5% in either group or ≥ 4% difference between groups)

System organ class	Adverse Events			
	Blonanserin (N = 156)		Risperidone (N = 145)	
Preferred term	n	%	n	%
Patients with events	153	98.1	143	98.6
Endocrine disorder				
Hyperprolactinemia	1	0.6	8	5.5
Gastrointestinal disorders				
Salivary hypersecretion	31	19.9	26	17.9
Constipation	16	10.3	21	14.5
Nausea	16	10.3	16	11.0
Vomiting	13	8.3	9	6.2
Diarrhea	12	7.7	13	9.0
General disorders and administration site conditions				
Malaise	27	17.3	34	23.4
Gait abnormal	27	17.3	22	15.2
Thirst	20	12.8	16	11.0
Asthenia	17	10.9	14	9.7
Difficulty in walking	12	7.7	17	11.7
Pyrexia	10	6.4	16	11.0
Infections and infestations				
Nasopharyngitis	27	17.3	28	19.3
Injury, poisoning and procedural complications				
Contusion	9	5.8	6	4.1
Excoriation	8	5.1	7	4.8
Investigations				
Blood prolactin increased	72	46.2	114	78.6
Blood creatine phosphokinase increased	23	14.7	16	11.0
Weight decreased	12	7.7	8	5.5
White blood cell count increased	8	5.1	9	6.2
Alanine aminotransferase increased	5	3.2	12	8.3
Aspartate aminotransferase increased	3	1.9	9	6.2
Weight increased	1	0.6	7	4.8
Gamma glutamyl transpeptidase increased	—	—	6	4.1
Metabolism and nutrition disorders				
Anorexia	19	12.2	23	15.9
Increased appetite	2	1.3	10	6.9
Musculoskeletal and connective tissue disorders				
Musculoskeletal stiffness	24	15.4	20	13.8
Nervous system disorders				
Bradykinesia	57	36.5	56	38.6
Tremor	49	31.4	36	24.8
Akathisia	45	28.8	25	17.2
Somnolence	32	20.5	29	20.0
Headache	24	15.4	21	14.5
Dizziness	20	12.8	16	11.0
Dyslalia	18	11.5	13	9.0
Hypokinesia	15	9.6	20	13.8
Dyskinesia	12	7.7	5	3.4
Dizziness postural	11	7.1	9	6.2
Psychiatric disorders				
Insomnia	66	42.3	52	35.9
Anxiety	27	17.3	18	12.4
Irritability	22	14.1	11	7.6
Excitability	18	11.5	7	4.8
Depression	10	6.4	13	9.0
Skin and subcutaneous tissue disorders				
Pruritus	10	6.4	2	1.4
Vascular disorders				
Orthostatic hypotension	1	0.6	7	4.8

Note

Adapted from Miura S. 2008, Table 11.

#### 3.3.2 Extrapyramidal symptoms

Extrapyramidal adverse events occurred in a similar incidence (about two‐thirds of the patients) in the two groups (Table 5). Common extrapyramidal adverse events were bradykinesia, tremor, akathisia, salivary hypersecretion, gait abnormal, musculoskeletal stiffness, dyslalia, and hypokinesia in each group (Table S5). Akathisia was more common in the blonanserin group.

The maximum change in DIEPSS total score excluding overall severity was about one in each group (Table S6). No major intergroup difference was found in the change at any evaluation point (Table S7).

The percentage of patients who concomitantly used any antiparkinsonian drugs decreased from baseline during the study in each group and was lower in the risperidone group than in the blonanserin group throughout the study (data not shown).

#### 3.3.3 Laboratory tests and other assessments

The incidence of abnormal change in prolactin notably differed in the two groups. Prolactin was elevated in both groups at baseline; during the study, the elevated prolactin worsened in the risperidone group and was returning to normal in the blonanserin group. No clinically relevant abnormal change was found in the other laboratory parameters including glucose metabolism parameters, body weight, vital signs, or ECG parameters (Tables S8‐S11).

## 4 DISCUSSION

Blonanserin showed similar efficacy against both positive and negative symptoms of schizophrenia to that of risperidone regardless of strategies for the handling of missing data. The robustness of the results was confirmed. Data from the risperidone group in this study are considered appropriate for the comparison because risperidone showed comparable efficacy in this study to that observed in previous phase 3 studies; the change in BPRS total score was −7.4 in this study and −7.2 in the previous study.^15^, ^16^, ^17^ The recent meta‐analysis of 167 randomized controlled trials indicated that blonanserin has similar efficacy in positive symptoms compared to other SGAs and potentially superior efficacy in negative symptoms to other SGAs.^2^ Blonanserin also exhibited comparable efficacy to other SGAs in another meta‐analysis of randomized controlled studies comparing blonanserin with other antipsychotics, most of which were conducted in Japan.^1^ The mean daily dose of blonanserin in the present study was 13.41 mg/d and is comparable to the dose in those randomized controlled studies.^1^

The incidences of prolactin increase, weight gain, and orthostatic hypotension were rarer for blonanserin, whereas those of akathisia and excitability were more common for blonanserin, compared with risperidone. Most of the adverse events were mild to moderate in severity in the both drugs. In general, dopamine is a prolactin inhibitor,^18^ which may result in amenorrhea or secondary decrease in bone mineral density in female patients and sexual dysfunction such as erectile disturbance in male patients.^19^, ^20^ Patients may hesitate to complain about such events to their physicians although the failure or delayed detection of the event may lead to serious consequences. Therefore, a relatively low risk of prolactin elevation would be an important factor in selecting antipsychotics. Weight gain is generally induced with most SGAs,^21^ but the risk is lower in blonanserin. The excitability increase in blonanserin group was likely due to low affinity to adrenaline α_1_ and histamine H_1_, resulting in blonanserin not being likely to cause excessive sedation. Orthostatic hypotension was more common in risperidone than in blonanserin, likely because risperidone is an α_1_‐adrenergic receptor blocker. Taken together, varied safety profiles of the two drugs were mainly related to the receptor‐binding profiles, suggesting varied, and potentially more beneficial, clinical features of blonanserin compared with risperidone.

None of the serious adverse events reported in the blonanserin group were related to treatment with blonanserin. The incidence of adverse events leading to drug discontinuation and the incidence of extrapyramidal symptoms were not markedly different for the two drugs. Reported extrapyramidal adverse events were similar in both groups such as bradykinesia, tremor, akathisia, salivary hypersecretion, and gait abnormal. Akathisia is one of the clinically significant side effects of antipsychotics that affect treatment adherence in patients with schizophrenia. The incidence of akathisia was 28.8% for blonanserin in the present study and 11.1% and 23.3% for olanzapine and aripiprazole, respectively, in Japanese 8‐week trials in patients with schizophrenia.^15^, ^16^ Although the incidence of akathisia was higher for blonanserin, it did not seem to affect treatment adherence; a Japanese long‐term study showed that the withdrawal rate due to adverse events or worsening of symptoms was as low as 6.9% during 28 weeks of treatment.^22^ Akathisia associated with blonanserin does not seem to pose an unacceptable risk as long as a patient is carefully observed primarily at the early stage of treatment or after dose increase with blonanserin, which would also be the case for other SGAs. In the updated meta‐analysis of 10 randomized controlled trials including this study, the overall safety outcome did not differ between blonanserin and other antipsychotics including risperidone or aripiprazole while some variation in each adverse event, such as akathisia, extrapyramidal symptoms, prolactin levels, or weight gain.^23^

Blonanserin has a functional selectivity with high affinity for the dopamine D_2_, D_3,_ and the serotonin 5‐HT_2A_ receptors over other receptors.^24^ Its higher affinity for D_2_ receptors than for 5‐HT_2A_ receptors improves various psychiatric symptoms in schizophrenia along with the fewer side effects such as extrapyramidal.^25^ Negative symptoms are often treatment resistant to some current antipsychotics.^26^ That may lead to low participation in psychosocial treatment and poor outcome of patients with schizophrenia. The current consensus on negative symptoms advocates that the each domain of negative symptoms, that is, blunted affect, alogia, asociality, anhedonia, and avolition, may have separate neurobiological substrates and may represent different therapeutic targets.^27^

Blonanserin was reportedly more effective to negative symptoms than haloperidol.^5^ The recent meta‐analysis indicated that blonanserin has potentially superior efficacy in negative symptoms to other SGAs.^2^ In our study, a similar tendency was observed; reduction of PANSS negative scores tended to be larger in blonanserin (−3.4 ± 4.6) than risperidone (−3.0 ± 4.3), though not significantly. It might be likely that the selective receptor‐binding affinity profile of blonanserin could contribute to improvement of negative symptoms in particular domains not affected by FGAs or other SGAs.

Cognitive impairments, as well as negative symptoms, are associated with diminished motivation and pleasure in patients with schizophrenia and hinder their personal recovery, which remain unmet medical needs in treatment of schizophrenia.^28^ It is of great interest to investigate whether the efficacy of blonanserin against negative symptoms could extend to cognitive impairments. Further studies are warranted to clarify the potential benefit of blonanserin which may provide a biological clue to the pathogenesis of negative and cognitive symptoms of schizophrenia and to determine the clinical role of blonanserin in the latest treatment of schizophrenia.

In participants enrolled in this study, 70% had chronic disorder that lasted for more than 5 years. Treatment‐naïve patients were about 10% of the sample, and negative symptoms are dominant rather than acute phase symptoms.

There are several methodological limitations to this study. Prior antipsychotics were not tapered off with placebo substitution, and patients were switched to a low dose of the study drug alone regardless of the dose of the prior drug. Patients receiving a high dose of prior antipsychotics at baseline might have experienced exacerbation of symptoms after initiation of study treatment. Placebo run‐in was not included in the study because placebo use in psychiatry was commonly considered unethical at the time of the study in Japan.

## 5 CONCLUSION

Blonanserin showed the similar efficacy to risperidone in both positive and negative symptoms in schizophrenia with a lower risk of prolactin increase, weight gain, and orthostatic hypotension compared with risperidone. Blonanserin will serve as a favorable treatment option for schizophrenia in daily clinical practice. Further investigation is needed to clarify the potential benefit of blonanserin in the context of the latest schizophrenia treatment.

## CONFLICT OF INTEREST

Philip D. Harvey has received lecture honoraria from Sumitomo Dainippon Pharma Co, Ltd within the last 3 years. Hiroshi Nakamura is the employee of Sumitomo Dainippon Pharma Co., Ltd. Additional details are to be described, once all COI disclosure forms are collected.

## AUTHOR CONTRIBUTIONS

SM took responsibility for the design, data collection, case handling, and interpretation of data as a chief investigator for the study. HN wrote the first draft of the manuscript including literature searches. PH finalized the manuscript. All authors had full access to all study data, had final responsibility for the decision to submit for publication, took part in either drafting or revising the manuscript, and approved the final version of the manuscript.

## DATA REPOSITORY

There are no data listings publicly available for this study, because information about public data sharing was not included in the informed consent form of this study.

## APPROVAL OF THE RESEARCH PROTOCOL BY AN INSTITUTIONAL REVIEWER BOARD

The study protocol was approved by the institutional review board of each study site.

## INFORMED CONSENT

All subjects (and/or their legal representatives if patients were unable to give consent or younger than 20 years old) provided written informed consent.

## REGISTRY AND THE REGISTRATION NO. OF THE STUDY/TRIAL

The study has not been registered in any publicly accessible database since the study was conducted before November 1st, 2013, when the study registration became mandatory.
