# Supplementary material for: Blonanserin vs risperidone in Japanese patients with schizophrenia: A post hoc analysis of a phase 3, 8‐week, multicenter, double‐blind, randomized controlled study
Source: Neuropsychopharmacol Rep. 2019 Dec 1;40(1):63–72. doi: 10.1002/npr2.12089 (PMC7292214; doi:10.1002/npr2.12089)
Supplement: Supplementary file 1 [file NPR2-40-63-s001.docx]

TABLE S1 Comparisons of CGI-I

for prior antipsychotics at baseline

|  | Blonanserin (N=156) | | Risperidone (N=144) | |
| --- | --- | --- | --- | --- |
|  | n | % | n | % |
| Very much improved | 1 | 0.7 | 0 | 0.0 |
| Much improved | 31 | 21.7 | 26 | 19.8 |
| Minimally improved | 76 | 53.1 | 69 | 52.7 |
| No change | 26 | 18.2 | 29 | 22.1 |
| Minimally worse | 3 | 2.1 | 6 | 4.6 |
| Much worse | 2 | 1.4 | 1 | 0.8 |
| Very much worse | 0 | 0.0 | 0 | 0.0 |
| Not Evaluable | 4 | 2.8 | 0 | 0.0 |
| Patients with Improvement (%)^†^ | 22.4 | | 19.8 | |
| Without prior therapy^‡^ | 13 | | 13 | |

Abbreviation: CGI-I, Clinical Global Impressions-Improvement

^†^Very much improved + much improved.

^‡^These patients were excluded in the denominator for calculating the percentage of patients with improvement.

TABLE S2 Comparisons of change in BPRS total score and

cluster scores at end of study

|  |  |  | Baseline | End of study | Change from baseline | |
| --- | --- | --- | --- | --- | --- | --- |
| Scale | Group | N | Mean±SD | Mean±SD | Mean±SD | 95% CI |
| Total | Blonanserin | 156 | 47.9±9.1 | 40.7±12.8 | −7.2±11.0 | −8.9, −5.5 |
|  | Risperidone | 144 | 47.9±10.0 | 40.5±13.2 | −7.4±11.2 | −9.3, −5.6 |
| Anxiety/depressive mood | Blonanserin | 156 | 9.8±3.7 | 8.2±3.8 | −1.6±3.2 | −2.1, −1.1 |
|  | Risperidone | 144 | 9.4±3.7 | 7.8±3.6 | −1.6±3.6 | −2.2, −1.0 |
| Emotional withdrawal | Blonanserin | 156 | 12.4±3.4 | 10.2±3.5 | −2.2±2.6 | −2.6, −1.8 |
|  | Risperidone | 144 | 12.7±3.3 | 10.8±3.6 | −2.0±2.7 | −2.4, −1.5 |
| Conceptual disorganization | Blonanserin | 156 | 11.3±3.5 | 9.9±4.0 | −1.4±3.2 | −2.0, −0.9 |
|  | Risperidone | 144 | 11.5±3.8 | 9.9±4.0 | −1.6±3.3 | −2.1, −1.1 |
| Excitement | Blonanserin | 156 | 7.2±2.3 | 6.3±2.7 | −1.0±2.4 | −1.3, −0.6 |
|  | Risperidone | 144 | 7.0±2.4 | 5.9±2.8 | −1.1±2.5 | −1.5, −0.7 |
| Hostility/suspiciousness | Blonanserin | 156 | 7.1±2.8 | 6.2±3.1 | −1.0±2.6 | −1.4, −0.6 |
|  | Risperidone | 144 | 7.2±3.0 | 6.0±3.1 | −1.2±2.9 | −1.7, −0.7 |

Abbreviation: BPRS, Brief Psychiatric Rating Scale

TABLE S3 Percentage of patients with CGI-I by evaluation point

| Evaluation point | Blonanserin | | Risperidone | |
| --- | --- | --- | --- | --- |
|  | N | Patients with improvement^†^  n (%) | N | Patients with improvement^†^  n (%) |
| Week 1 | 152 | 49 (32.2) | 139 | 38 (27.3) |
| Week 2 | 146 | 64 (43.8) | 132 | 59 (44.7) |
| Week 3 | 135 | 69 (51.1) | 125 | 63 (50.4) |
| Week 4 | 121 | 68 (56.2) | 116 | 66 (56.9) |
| Week 6 | 117 | 74 (63.2) | 113 | 72 (63.7) |
| Week 8 | 109 | 69 (63.3) | 108 | 73 (67.6) |

Abbreviation: CGI-I, Clinical Global Impressions-Improvement

^†^Very much improved + much improved.

TABLE S4 Comparison of final CGI-I

|  | Blonanserin (N=155) | | Risperidone (N=143) | |
| --- | --- | --- | --- | --- |
|  | N | % | n | % |
| Markedly improved | 27 | 17.4 | 21 | 14.7 |
| Moderately improved | 52 | 33.5 | 60 | 42.0 |
| Slightly improved | 48 | 31.0 | 33 | 23.1 |
| Unchanged | 14 | 9.0 | 20 | 14.0 |
| Slightly aggravated | 9 | 5.8 | 2 | 1.4 |
| Moderately aggravated | 4 | 2.6 | 5 | 3.5 |
| Markedly aggravated | 1 | 0.6 | 2 | 1.4 |
| Not Evaluable | 0 | 0.0 | 0 | 0.0 |
| Patients with Improvement^†^ |  | |  | |
| % | 51 | | 56.6 | |
| Intergroup difference^‡^ | −5.7, 16.9 | | | |

Abbreviation: CGI-I, Clinical Global Impressions-Improvement

^†^Very much improved + much improved.

^‡^95% CI (CI: confidence interval)

TABLE S5 Incidence of extrapyramidal adverse events

| **System organ class** | | **Blonanserin (N=156)** | | **Risperidone (N=145)** | |
| --- | --- | --- | --- | --- | --- |
|  | **Preferred term** | **n** | **%** | **N** | **%** |
| Patients with extrapyramidal adverse events | | 104 | 66.7 | 89 | 61.4 |
| Eye disorders | |  |  |  |  |
|  | Oculogyration | - | - | 1 | 0.7 |
| Gastrointestinal disorders | |  |  |  |  |
|  | Salivary hypersecretion | 31 | 19.9 | 26 | 17.9 |
|  | Dysphagia | 1 | 0.6 | 2 | 1.4 |
| General disorders and administration site conditions | |  |  |  |  |
|  | Gait abnormal | 27 | 17.3 | 22 | 15.2 |
|  | Difficulty in walking | 12 | 7.7 | 17 | 11.7 |
|  | Asthenia | 1 | 0.6 | 0 | 0 |
| Investigations | |  |  |  |  |
|  | Corneal reflex decreased | - | - | 1 | 0.7 |
| Musculoskeletal and connective tissue disorders | |  |  |  |  |
|  | Musculoskeletal stiffness | 22 | 14.1 | 19 | 13.1 |
|  | Posture abnormal | - | - | 1 | 0.7 |
| Nervous system disorders | |  |  |  |  |
|  | Bradykinesia | 56 | 35.9 | 55 | 37.9 |
|  | Tremor | 48 | 30.8 | 35 | 24.1 |
|  | Akathisia | 45 | 28.8 | 25 | 17.2 |
|  | Dyslalia | 18 | 11.5 | 12 | 8.3 |
|  | Hypokinesia | 15 | 9.6 | 20 | 13.8 |
|  | Dyskinesia | 12 | 7.7 | 5 | 3.4 |
|  | Dystonia | 7 | 4.5 | 4 | 2.8 |
|  | Speech disorder | 2 | 1.3 | 1 | 0.7 |
|  | Parkinsonian gait | 2 | 1.3 | 1 | 0.7 |
|  | Dysarthria | - | - | 1 | 0.7 |

TABLE S6 Maximum change from baseline in DIEPSS total score

excluding overall severity

|  |  | **Change from baseline** | | **Intergroup difference** | |
| --- | --- | --- | --- | --- | --- |
| **Group** | **N** | **Mean±SD** | **95% CI** | **Mean** | **95% CI** |
| Blonanserin | 156 | 1.3±2.7 | 0.9, 1.8 | −0.35 | −0.93, 0.24 |
| Risperidone | 145 | 1.0±2.4 | 0.6, 1.4 |  |  |

Abbreviations: CI, confidence interval; DIEPSS, Drug Induced Extra-Pyramidal Symptoms Scale; and SD, standard deviation.

TABLE S7 Change from baseline in DIEPSS total score

excluding overall severity by evaluation point

|  | **Blonanserin** | | **Risperidone** | |
| --- | --- | --- | --- | --- |
| **Evaluation point** | **N** | **Mean±SD** | **N** | **Mean±SD** |
| Week 1 | 152 | 0.1±1.3 | 140 | 0.1±1.5 |
| Week 2 | 146 | 0.1±2.2 | 132 | 0.0±1.5 |
| Week 3 | 135 | 0.3±2.5 | 124 | −0.2±1.8 |
| Week 4 | 122 | 0.3±2.8 | 116 | 0.0±2.0 |
| Week 6 | 117 | 0.3±3.0 | 113 | 0.1±2.5 |
| Week 8 | 109 | 0.0±2.6 | 108 | −0.1±2.2 |
| Last observation | 156 | 0.4±2.9 | 145 | 0.2±2.3 |

Abbreviations: DIEPSS, Drug Induced Extra-Pyramidal Symptoms Scale; and SD, standard deviation.

TABLE S8 Incidence of abnormal change in laboratory data and other parameters

| **Parameters** | | **Blonanserin** | | **Risperidone** | |
| --- | --- | --- | --- | --- | --- |
|  |  | **N** | **n (%)** | **N** | **n (%)** |
| Hematology | WBC count | 154 | 8 (5.2) | 143 | 9 (6.3) |
|  | RBC count | 154 | 2 (1.3) | 143 | 1 (0.7) |
|  | Hemoglobin | 154 | 1 (0.6) | 143 | 1 (0.7) |
|  | Hematocrit | 154 | 1 (0.6) | 143 | 1 (0.7) |
|  | Platelet count | 154 | 2 (1.3) | 143 | 0 (0.0) |
|  | Neutrophils | 123 | 2 (1.6) | 113 | 2 (1.8) |
|  | Neutrophils/stab leukocytes | 31 | 1 (3.2) | 30 | 0 (0.0) |
|  | Neutrophils/segmented leukocytes | 31 | 2 (6.5) | 30 | 2 (6.7) |
|  | Lymphocytes | 154 | 3 (1.9) | 143 | 5 (3.5) |
|  | Eosinophils | 154 | 1 (0.6) | 143 | 1 (0.7) |
|  | Basophils | 154 | 0 (0.0) | 143 | 0 (0.0) |
|  | Monocyte | 154 | 0 (0.0) | 143 | 0 (0.0) |
| Blood chemistry | AST (GOT) | 156 | 3 (1.9) | 144 | 9 (6.3) |
|  | ALT (GPT) | 156 | 5 (3.2) | 144 | 12 (8.3) |
|  | Alkaline Phosphatase | 156 | 0 (0.0) | 144 | 0 (0.0) |
|  | γ-GTP | 156 | 0 (0.0) | 144 | 6 (4.2) |
|  | Total protein | 156 | 1 (0.6) | 144 | 0 (0.0) |
|  | Total bilirubin | 156 | 4 (2.6) | 144 | 0 (0.0) |
|  | LDH | 156 | 6 (3.8) | 144 | 1 (0.7) |
|  | CPK | 156 | 23 (14.7) | 144 | 16 (11.1) |
|  | BUN | 156 | 4 (2.6) | 144 | 2 (1.4) |
|  | Creatinine | 156 | 0 (0.0) | 144 | 0 (0.0) |
|  | Total cholesterol | 156 | 5 (3.2) | 143 | 2 (1.4) |
|  | Triglyceride | 156 | 7 (4.5) | 143 | 4 (2.8) |
|  | Na | 156 | 2 (1.3) | 144 | 1 (0.7) |
|  | K | 156 | 4 (2.6) | 144 | 1 (0.7) |
|  | Cl | 156 | 2 (1.3) | 144 | 1 (0.7) |
|  | Prolactin | 156 | 74 (47.4) | 144 | 122 (84.7) |
|  | Phospholipid | 156 | 0 (0.0) | 144 | 3 (2.1) |
|  | Blood glucose | 156 | 3 (1.9) | 144 | 3 (2.1) |
|  | HbA1c | 156 | 0 (0.0) | 144 | 1 (0.7) |
|  | Insulin | 155 | 6 (3.9) | 144 | 5 (3.5) |
| Urinalysis | Urine glucose | 153 | 1 (0.7) | 143 | 0 (0.0) |
|  | Urine protein | 153 | 3 (2.0) | 143 | 0 (0.0) |
|  | Urine urobilinogen | 153 | 1 (0.7) | 143 | 0 (0.0) |
| Vital signs and weight | Supine blood pressure | 156 | 6 (3.8) | 144 | 4 (2.8) |
|  | Orthostatic blood pressure | 155 | 6 (3.9) | 144 | 7 (4.9) |
|  | Pulse rate | 156 | 5 (3.2) | 144 | 6 (4.2) |
|  | Body temperature | 156 | 14 (9.0) | 144 | 22 (15.3) |
|  | Weight | 155 | 13 (8.4) | 144 | 15 (10.4) |
| ECG | ECG findings | 156 | 13 (8.3) | 144 | 10 (6.9) |
|  | QT prolongation | 155 | 3 (1.9) | 144 | 2 (1.4) |
| EEG | | 60 | 2 (3.3) | 53 | 2 (3.8) |

Abbreviations: ECG, electrocardiography; and EEG, electroencephalography.

TABLE S9 Change from baseline in glucose metabolism parameters

| **Parameters** | **Group** | **Baseline** | | **End of study** | |
| --- | --- | --- | --- | --- | --- |
|  |  | **n** | **Mean±SD** | **n** | **Mean±SD** |
| Blood glucose (mg/dL) | Blonanserin | 154 | 89.6±10.6 | 140 | 89.5±14.7 |
|  | Risperidone | 141 | 88.6±10.6 | 136 | 89.5±10.5 |
| HbA1c (%) | Blonanserin | 156 | 4.81±0.41 | 154 | 4.82±0.43 |
|  | Risperidone | 144 | 4.83±0.43 | 144 | 4.85±0.41 |
| Insulin (μU/mL) | Blonanserin | 151 | 7.90±6.55 | 139 | 7.99±6.84 |
|  | Risperidone | 139 | 7.50±6.02 | 134 | 7.68±9.32 |

Abbreviations: SD, standard deviation.

TABLE S10 Change from baseline in weight

| **Group** | **Baseline** | | **End of study** | |
| --- | --- | --- | --- | --- |
|  | **N** | **Mean±SD** | **N** | **Mean±SD** |
| Blonanserin | 156 | 61.48±12.73 | 155 | 60.35±12.72 |
| Risperidone | 144 | 61.15±12.94 | 144 | 61.00±13.19 |

Abbreviations: SD, standard deviation.

Unit: kg.

TABLE S11 Change from baseline in QTc

| **Parameters**  **(method)** | **Evaluation point** | **Blonanserin** | | | **Risperidone** | | |
| --- | --- | --- | --- | --- | --- | --- | --- |
|  |  | **n** | **Mean±SD** | **95% CI** | **n** | **Mean±SD** | **95% CI** |
| QTcB^†^  (Visual) | Week 2 | 145 | −10.2**±**28.8 | −14.9, −5.5 | 130 | 1.0**±**29.1 | −4.0, 6.1 |
|  | Week 4 | 122 | −7.1**±**28.2 | −12.2, −2.1 | 114 | 1.0**±**27.1 | −4.0, 6.1 |
|  | Week 8 | 108 | −4.5**±**28.1 | −9.9, 0.8 | 105 | 1.0**±**28.5 | −4.5, 6.5 |
|  | End of study | 154 | −1.1**±**30.1 | −5.9, 3.7 | 144 | 0.1**±**28.5 | −4.6, 4.8 |
| QTcB^†^  (Tangent) | Week 2 | 145 | −11.7**±**30.5 | −16.7, −6.7 | 130 | 2.3**±**30.6 | −3.0, 7.6 |
|  | Week 4 | 122 | −7.1**±**30.9 | −12.7, −1.6 | 114 | 1.3**±**30.2 | −4.3, 6.9 |
|  | Week 8 | 108 | −4.6**±**29.6 | −10.3, 1.0 | 105 | 2.0**±**30.5 | −4.0, 7.9 |
|  | End of study | 154 | −0.7**±**31.5 | −5.7, 4.3 | 144 | 2.6**±**31.4 | −2.6, 7.8 |
| QTcF^‡^  (Visual) | Week 2 | 145 | −6.6**±**23.7 | −10.5, −2.8 | 130 | −1.5**±**23.6 | −5.6, 2.6 |
|  | Week 4 | 122 | −3.3**±**22.8 | −7.4, 0.8 | 114 | 0.3**±**22.3 | −3.9, 4.4 |
|  | Week 8 | 108 | −1.9**±**23.1 | −6.3, 2.5 | 105 | 2.4**±**22.8 | −2.0, 6.8 |
|  | End of study | 154 | −0.8**±**23.5 | −4.6, 2.9 | 144 | 0.3**±**23.4 | −3.6, 4.1 |
| QTcF^‡^  (Tangent) | Week 2 | 145 | −8.2**±**25.0 | −12.3, −4.1 | 130 | −0.3**±**24.4 | −4.5, 4.0 |
|  | Week 4 | 122 | −3.2**±**24.9 | −7.6, 1.3 | 114 | 0.7**±**24.5 | −3.8, 5.2 |
|  | Week 8 | 108 | −2.0**±**23.1 | −6.4, 2.4 | 105 | 3.4**±**24.9 | −1.4, 8.2 |
|  | End of study | 154 | −0.4**±**24.1 | −4.3, 3.4 | 144 | 2.7**±**26.0 | −1.6, 7.0 |

Abbreviations: CI, confidence interval; and SD, standard deviation.

^†^QT corrected with Bazett method.

^‡^QT corrected with Fridericia method.
